# The Effect of Intraoperative Magnesium Sulphate Infusion on Emergence Agitation after Ambulatory Ophthalmic Surgery in Children

**DOI:** 10.3390/jcm9124126

**Published:** 2020-12-21

**Authors:** Yea-Ji Lee, Bo-Young Kim, Jae-Hee Park, Sae-Yeon Kim, Hee-Yeon Park, Sang-Hwan Do

**Affiliations:** 1 Department of Anaesthesiology and Pain Medicine, Konkuk University Medical Centre, Seoul 05030, Korea; ladydaisy82@naver.com; 2 Department of Anaesthesiology and Pain Medicine, Seoul National University Bundang Hospital, Seongnam 13620, Korea; to123215@gmail.com (B.-Y.K.); cecillia2@gmail.com (S.-Y.K.); 3 Department of Anaesthesiology and Pain Medicine, Severance Hospital, Seoul 03722, Korea; sandman49@naver.com; 4 Department of Anaesthesiology and Pain Medicine, Gil Medical Centre, Gachon University College of Medicine, Incheon 21565, Korea; hypark@gilhospital.com; 5 Department of Anaesthesiology and Pain Medicine, Seoul National University College of Medicine, Seoul 03080, Korea

**Keywords:** ambulatory surgery, emergence agitation, magnesium sulphate, ophthalmic surgery, paediatric patients

## Abstract

This study investigated whether intraoperative infusion of magnesium sulphate reduces the incidence of emergence agitation (EA) in paediatric patients who undergo ambulatory ophthalmic surgery using the Paediatric Anaesthesia Emergence Delirium (PAED) scale. Ninety-two paediatric patients who were scheduled for elective ophthalmic surgery were randomly allocated to two groups: control or magnesium. In the magnesium group, patients received an initial intravenous loading dose of 30 mg/kg of 10% solution of magnesium sulphate over 10 min and then a continuous infusion of 10 mg/kg×h during the surgery. In the control group, an equal volume of 0.9% isotonic saline was administered in the same way as in the magnesium group. The PAED scale was assessed at 15-min intervals until the PAED score reached below 10 at the postanaesthetic care unit. EA was defined as a PAED score of 10 or higher. Of the 86 patients recruited, 44 and 42 were allocated to the control and magnesium groups, respectively. The incidence of EA was 77.3% in the control group and 57.1% in the magnesium group (odds ratio, 0.392; 95% confidence interval, 0.154 to 0.997; *p* = 0.046). The intraoperative infusion of magnesium sulphate significantly reduced the incidence of EA.

## 1. Introduction

Emergence agitation (EA) is one of the most common postoperative complications during recovery from general anaesthesia. EA is not only an unpleasant experience for parents and caregivers but also causes unexpected events, such as removal of drains or catheters, self-harm and delayed discharge of patients. The reported incidence of EA varies widely, ranging from 30% to 80% depending on definition, research background (patients’ age group, surgical site or type of surgery, etc.), assessment method and monitoring period used in each study [1,2,3]. While EA can occur in any age group, it is more commonly observed in children aged 3–9 years [2]. It has further been reported that the administration of sevoflurane and desflurane as general anaesthetics increases the onset of EA [4,5]. Ophthalmic and otorhinolaryngological surgeries are also considered risk factors for EA [6]. Although pain is known to be one of the risk factors of EA, it remains controversial whether pain is closely associated with the occurrence of EA [7,8,9,10]. The incidence of EA is reportedly 38% when sevoflurane is administered for minor procedures without any additional medication for pain [5].

Magnesium sulphate is increasingly used as an anaesthetic-sparing medication and analgesic adjuvant. A recent survey reported that intraoperative administration of magnesium sulphate to paediatric patients who underwent adenotosillectomy significantly reduced the incidence and the severity of EA without any adverse events or delay of recovery from general anaesthesia [3].

The present study explored the hypothesis that the administration of magnesium sulphate, relative to that of isotonic saline, would reduce the incidence and severity of EA in paediatric patients undergoing ophthalmic procedures performed in ambulatory settings.

## 2. Materials and Methods

### 2.1. Study Design, Participants and Recruitment

This prospective randomised, controlled, double-blinded study was approved by the Institutional Review Board of Seoul National University Bundang Hospital (B-1605/346-002). The study was registered at clinicaltrials.gov (NCT03208452, principal investigator: Yea-Ji Lee, date of registration: 6 March 2017). The study was performed at Seoul National University Bundang Hospital after obtaining verbal agreement from the patients and written informed consent from legal representatives (generally the patients’ parents). The present study abided by the Declaration of Helsinki.

We enrolled patients aged 4 to 7 with an American Society of Anesthesiologists physical status of I–II. All patients were scheduled to undergo elective strabismus surgery or epiblepharon repair. Exclusion criteria were as follows: abnormal electrolyte balance, cardiomyopathy, conduction disorder, myasthenia gravis or other neuromuscular diseases, impaired renal function, comatose status or patients who elected not to participate in this study.

### 2.2. Interventions and Procedures

Patients were randomised to receive either 10% solution of magnesium sulphate or 0.9% isotonic saline (placebo). Block randomisation (block size of four) was accomplished by a person who was not involved in the study using Random Allocation Software. The patients and the investigators who assessed postoperative outcomes were blinded to the group assignments.

In the pre-anaesthetic holding area, the patients and their parents received instructions as to how to proceed with the volatile induction and maintenance of anaesthesia (VIMA). Patients were accompanied by one of their parents when entering the operating room. On arrival at the operating room, routine monitoring including pulse oximetry, electrocardiography and noninvasive blood pressure measurement, were performed. The patients were encouraged to engage in spontaneous mask ventilation with oxygen and nitrous oxide (inspired oxygen fraction = 0.4) by a parent for a few minutes before the concentration of sevoflurane was increased gradually and the patients lost consciousness. Manual mask ventilation with 100% oxygen and 8 vol% of sevoflurane was then performed by an anaesthesiologist. During manual mask ventilation, an intravenous catheter was placed into the patient’s peripheral vein and a preoperative blood sample was collected. After administering 10 μg/kg dose of alfentanil, the LMA Flexible (Teleflex Medical, Co. Westmeath, Ireland) was inserted into the patient’s airway. Anaesthesia was maintained with 2.5–3 vol% of end-tidal sevoflurane concentration without the use of a neuromuscular blocking agent. The concentration of sevoflurane was titrated to maintain heart rate and mean arterial blood pressure within 20% of their baseline values. If these two values decreased below 20% of their baseline values, atropine or vasopressor was administered.

In the magnesium group, a dose of 30 mg/kg of 10% solution of magnesium sulphate was loaded intravenously over 10 min before surgery started, followed by the continuous infusion dose of 10 mg/kg×h during the surgery. In the control group, an equal volume of 0.9% isotonic saline was administered in the same manner as the magnesium. At the end of the surgery, a postoperative blood sample was obtained to measure the patient’s magnesium levels (the reference levels of magnesium in our institution are 0.45 to 0.6 mmol/L).

### 2.3. Outcomes

When patients were transferred to the postanaesthesia care unit (PACU), EA was assessed with the Paediatric Anaesthesia Emergence Delirium (PAED) scale (Table 1) [11]. The total score was obtained by summing the scores of each item. To establish good inter-rater reliability using the PAED scale, we designated only one PAED assessor. The assessor was trained by one of the authors during the pilot study in how to assess each of the five individual items of PAED scale. We made our own guidelines to score each item as objectively as possible and thus reduce intersubject variability and acquainted the assessor with these guidelines as part of the training process.

If the total score was 10 or higher, patients were considered to have developed EA [3,12,13]. Evaluation of the PAED scale was repeated at 15-min intervals until the score reached below 10. If the score was 10 or more, 0.5 μg/kg of fentanyl (maximum dose of 2 μg/kg) was administered. The perioperative adverse effects of intravenous magnesium sulphate, such as bradycardia, conduction disorders, cardiac arrest, hypotension, breathing difficulties or muscle weakness, were assessed.

The primary outcome assessed was the incidence of EA in the PACU. The secondary outcomes assessed were the calculated PAED score and the need for an additional dose of fentanyl.

### 2.4. Statistical Analysis

Previous studies reported that the incidence of EA in paediatric patients undergoing strabismus surgery ranges between 40% and 86% [14]. We assumed the average incidence of EA in this study to be 60%. Based on our a priori analysis, we presumed that a reduction of the incidence of more than 30% in the magnesium group would indicate a significant effect. Expecting an equal distribution between the two groups and a 10% dropout, a total of 92 subjects were required to achieve a power of 80% and type 1 error of 5%.

SPSS software (ver. 25.0, IBM Co., Armonk, NY, USA) was used for the statistical analysis and a *p* value < 0.05 was considered to indicate statistical significance. The normality of the data distribution was tested using the Shapiro–Wilk test (results not shown). Student’s *t*-test or the Mann–Whitney *U* test was used depending on the results of the normality test. Differences in the incidence of EA at each time point were compared using the Chi-squared test or Fisher’s exact test. The odds ratio (OR) and 95% confidence interval (CI) were calculated. All data were expressed as mean ± standard deviation (SD), median (range) or as numbers (%).

## 3. Results

**Figure 1:**
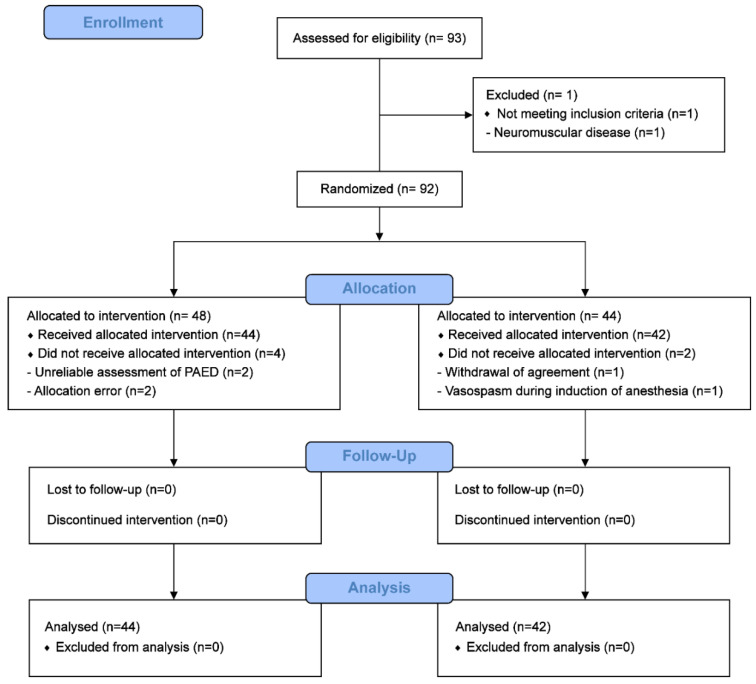
Consolidated Standards of Reporting Trials (CONSORT) diagram.

We initially assessed 93 patients for eligibility between March 2017 and December 2018. One patient with neuromuscular disease was excluded and the 92 remaining patients were randomised. After randomisation, four patients in the magnesium group and two patients in the control group were excluded. Finally, 86 patients (44 in the magnesium group and 42 in the control group) were included in analyses (Figure 1).

While the baseline characteristics of the patients were comparable between the groups, the postoperative serum ionised magnesium levels differed significantly between them (Table 2).

The incidence of EA at the PACU was significantly higher in the control group than in the magnesium group (77.3% vs. 57.1%; OR, 0.392; 95% CI, 0.154 to 0.997; *p* = 0.046). After 15 min of initial evaluation, EA persisted in nine patients in the control group and four in the magnesium group; however, there was no significant difference between the groups (26.5% vs. 16.7%; OR, 0.556; 95% CI, 0.149 to 2.075; *p* = 0.526). No patient showed EA after 30 min of initial evaluation (Table 3). There were significant differences in the severity of EA between the groups in the first evaluation (15.0 (10.0–17.0) vs. 11.5 (7.0–15.3); *p* = 0.019; control vs. magnesium). After 15 and 30 min, the severity of EA was similar in both groups (Table 3). The need for an additional dose of fentanyl was not significantly different between the two groups (Table 3). There were no perioperative side effects or delayed recovery associated with the administration of magnesium sulphate. The duration of PACU stay and total rescue dose of fentanyl did not differ significantly between the groups (Table 3). There was no incidence of adverse effects of magnesium sulphate in either group.

## 4. Discussion

This report demonstrates that the intraoperative infusion of magnesium sulphate significantly decreases the incidence and severity of EA in paediatric patients who undergo ambulatory ophthalmic surgery. Since we administered intravenous fentanyl to patients who developed EA, the administration of magnesium sulphate significantly prevented the need to administer a rescue dose of fentanyl at the first evaluation.

EA has been described as a mental and behavioural disturbance during recovery from general anaesthesia that may manifest as restlessness, agitation, inconsolable crying, disorientation, delusion and cognitive impairment [11]. While the aetiology of EA still remains unclear, some risk factors for EA have become generally accepted: preschool age, head and neck surgery, previous surgery, postoperative pain and inhalational agents associated with rapid awakening [14,15,16].

The relatively high incidence and severity of EA in both the control and magnesium groups can be explained by patients’ ages and the characteristics of the ophthalmic procedure. Preschool age has been considered an important factor associated with a high incidence of EA [8]. This may be ascribed to the psychological immaturity of preschool-aged patients and their inability to adapt to an unfamiliar environment upon their rapid emergence from sevoflurane anaesthesia. Undertaken to restore binocular single vision or for cosmetic reasons, strabismus surgery is one of the most common ophthalmic procedures performed on preschool-aged patients [14,17]. Epiblepharon is a common eyelid anomaly in Asian children that causes the eyelashes to turn inwards and which is addressed by making an adhesion between the anterior lamella of the eyelid and the lower eyelid retractors [18,19]. It is essential to apply ointment to the operation site and wear an eye patch after such ophthalmic procedures. These routine postsurgical steps may cause corneal irritation and visual disturbance, which can lead to patients developing agitation during recovery from general anaesthesia.

The rapid recovery from inhalational agents, especially sevoflurane and desflurane, has increasingly been considered a less important aetiological factor of EA. Delayed emergence from sevoflurane was not found to be associated with reduced EA [20]. Propofol-based anaesthesia provided a comparable emergence profile but induced a significantly lower incidence of EA [5,21]. In recent years, the excitatory effect of sevoflurane on the central nerve system has been increasingly ascribed to attendant elevated lactic acid concentrations in the brain [22]. In animal experiments, the administration of magnesium sulphate contributed to the suppression of lactic acid concentration and improvement of electroencephalographic changes in response to cerebral ischaemia [23,24].

NMDA (N-methyl-D-aspartate) receptor antagonists have analgesic, amnestic, muscle-relaxing properties that can help to protect airways [25,26]. In addition, NMDA receptor antagonists reportedly stimulate psychomotor activity, and lower doses of NMDA receptor antagonist (or mild NMDA receptor antagonist) helped to treat excess excitotoxicity [27,28,29]. Functioning as a gamma-aminobutyric acid (GABA_A_) receptor agonist, propofol blocks NMDA receptors in hippocampal neurons and may thus induce positive effects on postoperative mood [30].

An antagonist to the NMDA receptor, magnesium sulphate has been proposed as a general anaesthetic and has been found to enhance the activity of local anaesthetic agents [31,32,33]. In clinical trials, the administration of intravenous low-dose magnesium sulphate was associated with neuroprotective effects and a lower incidence of postoperative EA [34]. Glutamate, the major excitatory neurotransmitter in the central nervous system, is known to induce excitotoxicity by binding to the NMDA receptor; hence, NMDA receptor antagonists prevent excitotoxicity by attenuating glutamate release [27,30]. The neuroprotective potential of magnesium sulphate may be attributed to its antagonistic action to the NMDA receptors.

Despite the beneficial effects of perioperative magnesium sulphate on postoperative pain [35], the correlation between postoperative pain, magnesium sulphate and EA remains controversial. A previous meta-analysis reported that ketamine, α_2_-agonists and fentanyl were shown to be effective in the prevention of EA, but the effect was involved with mechanisms such as potentiation of anaesthesia or sedation rather than their analgesic properties [6]. Another meta-analysis demonstrated that while the administration of magnesium sulphate did not reduce postoperative pain, it significantly lowered the incidence of EA [36]. These findings suggest that magnesium sulphate diminishes EA by regulating neurotoxicity rather than through its analgesic effect.

Although opioids, in particular fentanyl, have demonstrated efficacy in the treatment and prevention of EA in children, they are associated with undesirable side effects such as respiratory depression, nausea, vomiting and delayed recovery [2,4,37]. Controversy concerning the use of magnesium sulphate persists due to its associated cardiovascular and neuromuscular effects. However, if magnesium sulphate is administered at an appropriate dose and with a suitable method, these adverse effects can be avoided [3,38,39]. Indeed, the present study observed comparable intraoperative mean heart rate and mean blood pressure between the two groups and no other adverse effects related to magnesium sulphate were observed without delayed discharge from the PACU. Besides the effects on EA, magnesium sulphate has another favourable effect on the prevention of postoperative nausea and vomiting (PONV) [40]. Strabismus surgery is associated with PONV, which occurs with an incidence of 60% to 70% [41]. Considering all these aspects, magnesium sulphate would be a more proper choice than opioids for postoperative management of patients who undergo ambulatory ophthalmic surgery.

Ambulatory surgeries are performed with the goal of promoting early recovery and minimal hospitalisation [42]. As these aims might be compromised by the adverse effects of opioids, the use of magnesium sulphate as an alternative could help to improve the management of EA in paediatric ambulatory ophthalmic surgery without prolonging PACU stays or resulting in unexpected hospitalisation.

This study has some limitations. Firstly, postoperative pain was not assessed in this study because we considered that it would be difficult for paediatric patients to distinguish between surgical pain and irritation due to ointment. However, if we had evaluated postoperative pain, it might have been possible to provide further evidence to account for the relation between postoperative pain, magnesium sulphate and EA. Secondly, satisfaction questionnaires for patients’ parents or nursing staff were not administered. The reunion of patients and their parents in the PACU is not routine in our institution. For this reason, we decided not to include satisfaction survey questionnaires for parents in this study. Taking into account the positive effect of intraoperative infusion of magnesium on the incidence and severity of EA, we inferred that the procedure could reduce the work load of nursing staff in the PACU. However, if questionnaires had been provided, we could have demonstrated the clinical relevance of the infusion of magnesium for EA more completely.

## 5. Conclusions

Compared to the administration of 0.9% isotonic saline, the intraoperative infusion of magnesium sulphate significantly reduced the incidence and severity of EA following ophthalmic surgery. Magnesium sulphate can be delivered safely to paediatric patients without inducing adverse events or complications.

## Figures and Tables

**Table 1:** The Paediatric Anaesthesia Emergence Delirium (PAED) scale.

Item
A. The child makes eye contact with caregiver
B. The child’s actions are purposeful
C. The child is aware of his/her surroundings
D. The child is restless
E. The child is inconsolable

Items A, B and C were reverse scored: 4 = not at all, 3 = just a little, 2 = quite a bit, 1 = very much, 0 = extremely. Items D and E were scored as follows: 0 = not at all, 1 = just a little, 2 = quite a bit, 3 = very much, 4 = extremely.

**Table 2:** Patient characteristics and diagnoses.

	Control (*n* = 44)	Magnesium (*n* = 42)	*p* Value
Male, *n* (%)	25 (56.8)	16 (38.1)	0.082
Age (year)	5 (4–6)	5 (4–6)	0.548
Weight (kg)	19.1 (16.8–23.7)	21.3 (18.6–26.0)	0.136
Preop. Mg (mmol/L)	0.6 ± 0.1	0.5 ± 0.1	0.321
Postop. Mg (mmol/L)	0.5 (0.5–0.5)	0.8 (0.7–0.9)	<0.001 *
Intraoperative MBP (mmHg)	60 ± 9	60 ± 9	0.953
Intraoperative MHR (bpm)	101 ± 14	98 ± 14	0.396
ASA status, *n* (%)			1.000
ASA I	41 (93.2)	39 (92.9)	
ASA II	3 (6.8)	3 (7.1)	
Anaesthesia time (min)	40 (30–45)	35 (30–46)	0.456
Operation time (min)	20 (20–30)	20 (15–31)	0.471
Mean sevo. (vol%)	3.0 (3.0–3.0)	3.0 (3.0–3.0)	0.816

Values indicate number of patients (%), median (range) or mean ± SD. Preop. Mg, preoperative serum ionized magnesium level; Postop. Mg, postoperative serum ionized magnesium level; MBP, mean blood pressure; MHR, mean heart rate; bpm, beats per minute; ASA, American Society of Anesthesiologists; Mean sevo., mean end-tidal concentration of sevoflurane. *: *p* <0.05. Chi-squared test, Fisher’s exact test, Student’s *t*-test or Mann–Whitney *U* test were used depending on whether a normal distribution was confirmed.

**Table 3:** Postoperative emergence agitation at PACU.

	Control (*n* = 44)	Magnesium (*n* = 42)	*p* Value
Incidence of EA, *n* (%)			
PAED 0	34 (77.3)	24 (57.1)	0.046 *
PAED 15	9 (20.5)	4 (9.5)	0.526
PAED 30	0 (0)	0 (0)	1.0
PAED scale			
PAED 0	15.0 (10.0–17.0)	11.5 (7.0–15.3)	0.019 *
PAED 15	7.3 ± 3.9	6.8 ± 2.6	0.559
PAED 30	6.8 ± 1.6	7.3 ± 1.0	0.592
Total rescue dose of fentanyl (μg)	12.0 (6.5–18.0)	9.8 (0–15.5)	0.141
PACU time (min)	31 (28–35)	30 (27–36)	0.990

Values indicate number of patients (%), median (range) or mean ± SD. PACU, postanaesthesia care unit; EA, emergence agitation; PAED 0, the first PAED score measured within 10 min from the end of anaesthesia; PAED 15, 15 min after PAED 0; PAED 30, 30 min after PAED 0. *: *p* < 0.05. Student’s *t*-test, Mann–Whitney *U* test, Chi-squared test or Fisher’s exact test were used depending on whether a normal distribution was confirmed.

## References

[1] Cole J.W., Murray D.J., McAllister J.D., Hirshberg G.E. (2002). Emergence behaviour in children: Defining the incidence of excitement and agitation following anaesthesia. Paediatr. Anaesth..

[2] Kanaya A. (2016). Emergence agitation in children: Risk factors, prevention, and treatment. J. Anesth..

[3] Abdulatif M., Ahmed A., Mukhtar A., Badawy S. (2013). The effect of magnesium sulphate infusion on the incidence and severity of emergence agitation in children undergoing adenotonsillectomy using sevoflurane anaesthesia. Anaesthesia.

[4] Costi D., Cyna A.M., Ahmed S., Stephens K., Strickland P., Ellwood J., Larsson J.N., Chooi C., Burgoyne L.L., Middleton P. (2014). Effects of sevoflurane versus other general anaesthesia on emergence agitation in children. Cochrane Database Syst. Rev..

[5] Uezono S., Goto T., Terui K., Ichinose F., Ishguro Y., Nakata Y., Morita S. (2000). Emergence agitation after sevoflurane versus propofol in pediatric patients. Anesth. Analg..

[6] Dahmani S., Stany I., Brasher C., Lejeune C., Bruneau B., Wood C., Nivoche Y., Constant I., Murat I. (2010). Pharmacological prevention of sevoflurane- and desflurane-related emergence agitation in children: A meta-analysis of published studies. Br. J. Anaesth..

[7] Abu-Shahwan I., Chowdary K. (2007). Ketamine is effective in decreasing the incidence of emergence agitation in children undergoing dental repair under sevoflurane general anesthesia. Paediatr. Anaesth..

[8] Aono J., Ueda W., Mamiya K., Takimoto E., Manabe M. (1997). Greater incidence of delirium during recovery from sevoflurane anesthesia in preschool boys. Anesthesiology.

[9] Cravero J.P., Beach M., Thyr B., Whalen K. (2003). The effect of small dose fentanyl on the emergence characteristics of pediatric patients after sevoflurane anesthesia without surgery. Anesth. Analg..

[10] Davis P.J., Cohen I.T., McGowan F.X., Latta K. (1994). Recovery characteristics of desflurane versus halothane for maintenance of anesthesia in pediatric ambulatory patients. Anesthesiology.

[11] Sikich N., Lerman J. (2004). Development and psychometric evaluation of the pediatric anesthesia emergence delirium scale. Anesthesiology.

[12] Aouad M.T., Yazbeck-Karam V.G., Nasr V.G., El-Khatib M.F., Kanazi G.E., Bleik J.H. (2007). A single dose of propofol at the end of surgery for the prevention of emergence agitation in children undergoing strabismus surgery during sevoflurane anesthesia. Anesthesiology.

[13] Bong C.L., Ng A.S. (2009). Evaluation of emergence delirium in Asian children using the Pediatric Anesthesia Emergence Delirium Scale. Paediatr. Anaesth..

[14] Kim J., Kim S.Y., Lee J.H., Kang Y.R., Koo B.N. (2014). Low-dose dexmedetomidine reduces emergence agitation after desflurane anaesthesia in children undergoing strabismus surgery. Yonsei Med. J..

[15] Voepel-Lewis T., Malviya S., Tait A.R. (2003). A prospective cohort study of emergence agitation in the pediatric postanesthesia care unit. Anesth. Analg..

[16] Veyckemans F. (2001). Excitation phenomena during sevoflurane anaesthesia in children. Curr. Opin. Anaesthesiol..

[17] Mizrak A., Erbagci I., Arici T., Ozcan I., Ganidagli S., Tatar G., Oner U. (2010). Ketamine versus propofol for strabismus surgery in children. Clin. Ophthalmol..

[18] Shin D.H., Woo K.I., Kim Y.D. (2017). Relationship between lower eyelid epiblepharon and epicanthus in Korean children. PLoS ONE.

[19] Woo K.I., Yi K., Kim Y.D. (2000). Surgical correction for lower lid epiblepharon in Asians. Br. J. Ophthalmol..

[20] Oh A.Y., Seo K.S., Kim S.D., Kim C.S., Kim H.S. (2005). Delayed emergence process does not result in a lower incidence of emergence agitation after sevoflurane anesthesia in children. Acta Anaesthesiol. Scand..

[21] Cohen I.T., Finkel J.C., Hannallah R.S., Hummer K.A., Patel K.M. (2003). Rapid emergence does not explain agitation following sevoflurane anaesthesia in infants and children: A comparison with propofol. Paediatr. Anaesth..

[22] Jacob Z., Li H., Makaryus R., Zhang S., Reinsel R., Lee H., Feng T., Rothman D.L., Benveniste H. (2012). Metabolomic profiling of children’s brains undergoing general anesthesia with sevoflurane and propofol. Anesthesiology.

[23] Bariskaner H., Ustun M.E., Ak A., Yosunkaya A., Ulusoy H.B., Gurbilek M. (2003). Effects of magnesium sulfate on tissue lactate and malondialdehyde levels after cerebral ischemia. Pharmacology.

[24] Lin J.Y., Chung S.Y., Lin M.C., Cheng F.C. (2002). Effects of magnesium sulfate on energy metabolites and glutamate in the cortex during focal cerebral ischemia and reperfusion in the gerbil monitored by a dual-probe microdialysis technique. Life Sci..

[25] Pender J.W. (1972). Dissociative anesthesia. Calif. Med..

[26] White P.F., Way W.L., Trevor A.J. (1982). Ketamine--its pharmacology and therapeutic uses. Anesthesiology.

[27] Gardoni F., Di Luca M. (2006). New targets for pharmacological intervention in the glutamatergic synapse. Eur. J. Pharmacol..

[28] Raffa R.B., Ortegón M.E., Robisch D.M., Martin G.E. (1989). In vivo demonstration of the enhancement of MK-801 by L-glutamate. Life Sci..

[29] Schmidt W.J., Bury D. (1988). Behavioural effects of N-methyl-D-aspartate in the anterodorsal striatum of the rat. Life Sci..

[30] Orser B.A., Bertlik M., Wang L.Y., MacDonald J.F. (1995). Inhibition by propofol (2,6 di-isopropylphenol) of the N-methyl-D-aspartate subtype of glutamate receptor in cultured hippocampal neurones. Br. J. Pharm..

[31] Coutinho E.M. (1966). Calcium, magnesium, and local anesthesia. J. Gen. Physiol..

[32] Meltzer S.J., Lucas D.R. (1907). Physiological and Pharmacological Studies of Magnesium Salts: V. the Influence of Nephrectomy Upon Their Toxicity. J. Exp. Med..

[33] Peck C.H., Meltzer S.J. (1916). Anesthesia in Human Beings by Intravenous Injection of Magnesium Sulphate. J. Am. Med. Assoc..

[34] Bilotta F., Gelb A.W., Stazi E., Titi L., Paoloni F.P., Rosa G. (2013). Pharmacological perioperative brain neuroprotection: A qualitative review of randomized clinical trials. Br. J. Anaesth..

[35] De Oliveira G.S., Castro-Alves L.J., Khan J.H., McCarthy R.J. (2013). Perioperative systemic magnesium to minimize postoperative pain: A meta-analysis of randomized controlled trials. Anesthesiology.

[36] Xie M., Li X.-k., Peng Y. (2017). Magnesium sulfate for postoperative complications in children undergoing tonsillectomies: A systematic review and meta-analysis. J. Evid. Based Med..

[37] Bortone L., Bertolizio G., Engelhardt T., Frawley G., Somaini M., Ingelmo P.M. (2014). The effect of fentanyl and clonidine on early postoperative negative behavior in children: A double-blind placebo controlled trial. Paediatr. Anaesth..

[38] Honarmand A., Safavi M., Badiei S., Daftari-Fard N. (2015). Different doses of intravenous Magnesium sulfate on cardiovascular changes following the laryngoscopy and tracheal intubation: A double-blind randomized controlled trial. J. Res. Pharm. Pract..

[39] Taheri A., Haryalchi K., Mansour Ghanaie M., Habibi Arejan N. (2015). Effect of low-dose (single-dose) magnesium sulfate on postoperative analgesia in hysterectomy patients receiving balanced general anesthesia. Anesth. Res. Pract..

[40] Ryu J.H., Sohn I.S., Do S.H. (2009). Controlled hypotension for middle ear surgery: A comparison between remifentanil and magnesium sulphate. Br. J. Anaesth..

[41] Ecoffey B. (2002). Recovery and outcome after minor pediatric surgery. Minerva Anestesiol..

[42] Pereira L., Figueiredo-Braga M., Carvalho I.P. (2016). Preoperative anxiety in ambulatory surgery: The impact of an empathic patient-centered approach on psychological and clinical outcomes. Patient Educ. Couns..

